# Profiling influences of senescent and aged fibroblasts on prostate carcinogenesis

**DOI:** 10.1038/sj.bjc.6604087

**Published:** 2008-01-08

**Authors:** J P Dean, P S Nelson

**Affiliations:** 1 Divisions of Human Biology and Clinical Research, Fred Hutchinson Cancer Research Center, Seattle, WA 98109, USA

**Keywords:** prostate, gene expression, senescence, paracrine, carcinogenesis

## Abstract

Experimental evidence suggests that ageing-associated alterations in the tissue microenvironment act to promote prostate carcinogenesis. In this review, we survey the cellular state of senescence, review its causes, and describe associations with ageing and cancer. We further discuss senescent stromal gene expression changes, which may mediate these effects, and that may serve as therapeutic targets.

In the United States and United Kingdom, prostate cancer is the most common non-cutaneous malignancy and the second leading cause of cancer deaths in men. In 2007, it is estimated that upwards of 200 000 and 30 000 new cases of prostate cancer will be diagnosed in the United States and United Kingdom, respectively, with more than 27 000 deaths predicted to be attributable to this disease in the United States alone (Office of National Statistics, 2006; Jemal *et al*, 2007). As men age, the risk of clinically detected prostate cancer increases dramatically from 0.01% for men aged less than 40 to about 14% for men aged 70 and older for a cumulative lifetime risk of 17% (Jemal *et al*, 2007). Autopsy studies designed to assess subclinical disease in different age cohorts report substantially higher rates of cancer incidence that parallel the age-associated increases in clinical diagnoses. For example, 27% of men aged 30–39 and 34% of men aged 40–49 harbour foci of adenocarcinoma in the prostate (Sakr *et al*, 1993). In fact, advancing age is consistently the greatest risk factor for the development of prostate cancer across racial and ethnic groups.

Historically, the relationship between ageing and carcinogenesis has been attributed to somatic mutations that accumulate in the epithelium over time due to oxidative damage to DNA nucleotide structure and due to failures of DNA damage repair systems. At the cellular level, increasing dysfunction with cumulative damage results in one of the following several cellular responses: cell-cycle arrest and injury repair, apoptosis, mitotic catastrophe, cellular senescence, or neoplastic transformation (Roninson, 2003). However, in addition to the epithelium, ageing-related cellular changes also impact the other tissue compartments that include extracellular matrix, infiltrating inflammatory cells, and constituent mesenchymal cell types such as fibroblasts and smooth muscle. Importantly, the microenvironment is increasingly recognised as an important contributor to the development of epithelial cancers (Krtolica *et al*, 2001; te Poele *et al*, 2002; Cunha *et al*, 2003) (Figure 1). In this context, the summation of cellular responses to the cumulative effects of metabolic stress and damage responses that occur during normal or accelerated ageing processes alters the microenvironment of the organ, and may contribute to the increased risk of carcinoma with advancing age. This review will discuss several lines of experimental evidence implicating ageing-related changes in fibroblast constituents of the prostate microenvironment as direct contributors to the process of carcinogenesis.

## CELLULAR SENESCENCE AND AGEING

**Figure 1:**
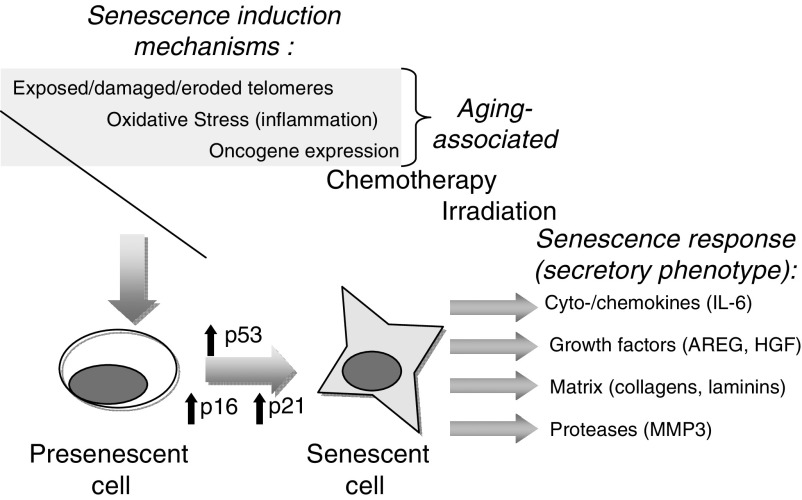
Ageing, senescence, and cellular phenotypic responses. With chronological ageing, telomere attrition leading to replicative exhaustion and/or an accumulation of environmental exposures leads to cellular senescence. DNA damage is sensed by the cell resulting in the activation of the p53–p21 and/or pRB–p16 pathways, leading to induction and maintenance of the senescent cellular state. The senescence programme consists of the robust expression of a diverse set of genes, a subset of which have the potential to alter the tissue microenvironment and promote carcinogenesis.

The term *senescence* is derived from the Latin word *senex*, meaning old man or old age. The concept of organismal senescence is used to generally describe the chronological ageing of whole organisms and ageing is commonly used interchangeably with senescence. The concept of cellular senescence is defined more precisely due to an improved understanding of the molecular events involved in this process. Cellular senescence was first associated with the replicative exhaustion, where normal diploid differentiated cells lose the ability to divide and enter a state of permanent growth arrest in the G_1_/G_0_ cell-cycle phase (Dimri *et al*, 1995; Roninson, 2003; Zhang, 2007). Importantly, senescent cells are not inert, as they remain metabolically active and are resistant to apoptosis, though they do not respond to mitogens. Upon becoming senescent, cells exhibit a classic morphology including enlargement with splaying and increased granularity. Classically identified in cell culture systems upon serial passage, replicative senescence has subsequently been attributed to telomere shortening. The cellular programme of senescence is subsequently activated by sensing of the altered telomeric structure. Activation of p53 and p21 has been implicated in the cell-cycle arrest of senescence. Additionally, inactivation of pRB and activation of p16 can also prevent cell-cycle progression (Roninson, 2003; Zhang, 2007) (Figure 1).

The senescence programme may also be engaged by other forms of cellular damage, including (a) cell stress (such as oxidative stress); (b) DNA damage; and (c) oncogene activation (e.g., K-ras, BRAF). Examples of DNA damage that induce senescence include chemotherapy and ionizing radiation. Interestingly, although the cell-cycle consequences of senescence are universal, the mechanisms that underlie the state of growth arrest and the gene expression programmes reflecting these varied mechanisms exhibit substantial differences (Zhang, 2007). Paradoxically, although it is thought that the cellular senescence programme may have developed as a mechanism to prevent malignant transformation by preventing continued replication in the setting of carcinogenic events (Roninson, 2003; Parrinello *et al*, 2005), accumulating data suggest that over time, residual senescent cells may alter the microenvironment to promote carcinogenesis in remaining non-senescent epithelium. This scenario has been suggested to represent an example of evolutionary antagonistic pleiotropy whereby natural selection would retain a mechanism (senescence) that protected the organism from a detrimental event (carcinogenesis), but this selected mechanism could have adverse consequences after selective pressures were no longer operative (Krtolica *et al*, 2001). In this example, senescence would be under strong positive selection during the reproductive phase of the life cycle, but pressures to avoid detrimental effects of senescence after reproductive years would not be propagated. As resident members of the prostate microenvironment, senescent epithelial or stromal cells (e.g. fibroblasts) could both contribute detrimental influences on neighbouring proliferating epithelium.

Consistent with the expectation of age-associated accrual of cellular damage, senescent cells accumulate with ageing. Senescence-associated *β*-galactosidase (SA-*β*-Gal), a metabolic marker primarily associated with senescence *in vitro*, has been used to identify increased numbers of senescent cells in aged human liver (Paradis *et al*, 2001) and skin (Dimri *et al*, 1995) compared to young tissues. Recently, other markers such as p16, *γ*-H2AX, and 53BP1 that are associated with *in vitro* senescence have been shown to accumulate with advancing age in a number of organisms that include mice (Krishnamurthy *et al*, 2004), primates (Herbig *et al*, 2006), and humans (Melk *et al*, 2004). These studies have provided clear molecular evidence that cells rendered senescent by ageing, stress, or oncogenesis accumulate over time.

## DIFFERENTIAL CONSEQUENCES OF SENESCENCE

The induction of senescence in the epithelium of tissues and organs such as the prostate gland is believed to have evolved as an intrinsic tumour suppressive mechanism in proliferative cells that accumulate DNA lesions potentially leading to neoplasia if not restrained. In a mouse model of prostate cancer, inhibition of *Pten*-associated carcinogenesis required functional p53 that operated, in part, through induction of senescence in the prostate epithelium (Chen *et al*, 2005). Analyses of human prostate cancer tissue supported this model, as senescent cells were identified in areas of benign prostatic hyperplasia and prostatic intraepithelial neoplasia (PIN), but were not observed within invasive neoplasms (Chen *et al*, 2005). Similarly, senescent cells have been shown to be relatively abundant in benign human nevi expressing the BRAF(V600E) oncogene (Michaloglou *et al*, 2005). These preneoplastic lesions are stable for decades before progressing to melanoma, at which point, senescent cells are infrequent. Mouse models of skin cancer, pancreatic cancer, and lung cancer have also been shown to contain senescent cells in preneoplastic lesions, but not in invasive cancers (Collado *et al*, 2005). These data indicate that a prerequisite for invasive neoplasia involves circumventing the growth-suppressing pathways that comprise the senescence programme. Interestingly, in a mouse model of oncogene-induced lymphoma, tumours capable of undergoing cellular senescence had a significantly better response to chemotherapy than tumours, which were not capable of senescence (Schmitt *et al*, 2002). However, these mice eventually succumbed to their lymphomas, demonstrating the limitations of the tumour-suppressive effects of the senescence pathway. *In vitro*, cancer cells that have undergone chemotherapy-induced senescence are (infrequently) able to resume proliferation (Roberson *et al*, 2005). This may suggest that senescent cancer cells might represent a potential reservoir for resistance and relapse to conventional medical therapies.

In contrast to the tumour-suppressing effects of the senescence programme operating within the individual cells harbouring oncogenic alterations as described above, senescent cells, particularly of the fibroblastic stromal compartment, may provide signals to neighbouring cells that promote carcinogenesis and tumour progression. For example, senescent but not pre-senescent breast fibroblasts were shown to transform immortal, non-neoplastic breast epithelial cell lines in a mouse xenograft model and to promote progression of neoplastic cell lines (Krtolica *et al*, 2001). Senescent fibroblasts also inhibited the normal differentiation programme of mammary gland development (Parrinello *et al*, 2005). Bavik *et al* (2006) extended these observations made in breast cancer to studies of prostate cancer and demonstrated that senescent prostate fibroblasts can stimulate the growth of pre-neoplastic and neoplastic prostate epithelium *in vitro*. An important study demonstrating the potential *in vivo* relevance of these observations focused on the localised regions directly surrounding human ovarian cancers. Increased numbers of senescent stromal cells were enriched in regions juxtaposed to cancer epithelium, a finding that indicates an intimate relationship between the influences and interactions of tumour cells and their attendant microenvironment (Yang *et al*, 2006).

## THE PROSTATE MICROENVIRONMENT AND PROSTATE CARCINOGENESIS

**Figure 2:**
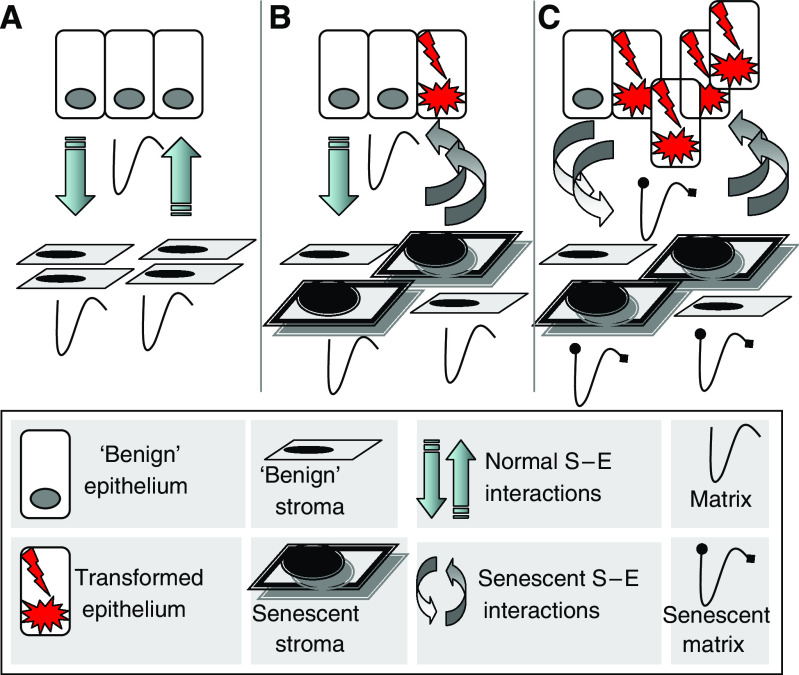
The prostate microenvironment and influence of senescence on carcinogenesis. (**A**) The normal microenvironment involves reciprocal paracrine interactions between epithelium and constituents of the stroma (fibroblasts, smooth muscle, endothelium, matrix, and inflammatory cells (not shown)) that serve to maintain homoeostasis and a functional differentiated tissue state. Microenvironmental signalling maintains tissue homoeostasis. (**B**) Cellular damage in the context of chronological ageing and/or oxidative stress leads to the accumulation of senescent stromal cells, which produce paracrine-acting factors capable of inducing or promoting epithelial carcinogenesis. (**C**) Continued promotion of carcinogenesis by the altered microenvironment, including changes in the matrix components, contributes to invasive and metastatic epithelial cell phenotypes. Aberrant paracrine signals from initiated epithelial cells also contribute to further stromal senescence and/or the development of a reactive stroma that further influences carcinogenesis.

The microenvironment of the prostate gland consists of several distinct cellular components, including epithelium, smooth muscle, fibroblasts, vascular structures, extravasated blood cells, as well as insoluble matrix and circulating soluble factors, all interacting in the context of an extracellular matrix. Although mutations have historically been viewed as the *sine qua non* of epithelial carcinogenesis, increasing evidence has demonstrated that alterations in the composition of the tumour microenvironment have the potential to contribute to the development and progression of invasive epithelial cancers (Figure 2). An example of this process was definitively shown in a mouse model, whereby alterations in TGF-*β* signalling in stromal fibroblasts produced forestomach neoplasms and PIN (Bhowmick *et al*, 2004).

The stroma of the prostate has been established to be an important regulator of proper gland morphogenesis and mature function. For example, recombination studies with mouse embryonic tissues have demonstrated that early androgen signalling occurs through the stromal compartment. This interaction was established through elegant experiments where AR-defective Tfm stromal tissues were not able to mediate prostatic epithelial development, while AR-defective epithelium combined with mesenchyme expressing a wild-type AR proceeded to develop normally (Cunha *et al*, 2003). In addition to the influence of stroma on developmental processes, the role of stromal cell phenotypes in prostate carcinogenesis has been investigated by several groups. Rowley and co-workers (Tuxhorn *et al*, 2002), determined that a reactive stroma exhibiting features associated with wound healing is capable of stimulating prostate epithelial proliferation *in vivo*. Many of these reactive characteristics have been observed in stroma directly associated with human cancers. Prostate fibroblasts derived from regions harbouring invasive cancer, termed cancer-associated fibroblasts, but not fibroblasts adjacent to normal epithelium, termed normal-associated fibroblasts, can promote the invasive phenotype of initiated prostate epithelial cells (Cunha *et al*, 2003). Finally, embryonal prostate stroma (urogenital mesenchyme), genetically programmed through exposure to testosterone and estradiol, is capable of promoting tumour development from immortal, but non-neoplastic prostate epithelium (Cunha *et al*, 2003). Thus, a neoplastic environment is capable of imparting a sustained cancer-promoting phenotype within cells comprising the peri-tumoural stroma. Senescent stromal cells may contribute to these tumour-promoting events. Currently, the identification and modulation of effector proteins derived from reactive and senescent stromal phenotypes is an active area of investigation.

## MEDIATORS OF EPITHELIAL CARCINOGENESIS IN THE AGED OR SENESCENT MICROENVIRONMENT

The demonstration that senescent cells accumulate in stromal compartments of aged individuals, coupled with experiments establishing the ability of senescent fibroblasts to profoundly alter the growth characteristics of epithelial cells, has led to efforts designed to define the specific molecular mechanisms by which this process occurs. Stroma-derived factors with the potential to influence epithelial phenotypes include cell surface molecules, secreted soluble factors, and secreted extracellular matrix proteins. To identify senescence- or ageing-associated changes in such factors, several groups have employed global profiling strategies in relatively unbiased searches. In a study reported by Begley *et al* (2005), microarrays were used to identify transcript alterations that specifically associated with differences in the age of cells comprising human prostate stroma. Primary fibroblast cultures were established from prostates resected from men aged 40, 40, 51, 52, 64, and 71 years. Differences in gene expression between younger men (aged 40–52) and older men (64–70) were compared to identify ageing-associated gene expression changes. Functionally, the stroma derived from older donors were found to be more permissive for the growth of the initiated, pre-neoplastic prostate epithelial cell line N15C6. Fifty-four unique transcripts were found to be differentially expressed with ageing, of which 41 were upregulated and 13 were downregulated. Of the upregulated transcripts, nine encoded secreted proteins. CXCL12 (SDF-1) was the most highly upregulated gene. Subsequent experiments confirmed age-associated increases in mRNA and protein secretion levels, as well as the contribution of increased CXCL12 signalling to increased epithelial proliferation (Begley *et al*, 2005).

Bavik *et al* (2006) reported the use of microarray profiling to identify transcript alterations in prostate fibroblasts that associated with the induction of senescence (Table 1). A comparison of three different senescence data sets identified 710 genes with consistent and significant alterations in gene expression of ⩾2-fold. Of these, 407 had increased expression levels with senescence and 303 were decreased. Seventy-one of the upregulated genes exhibited features of extracellular proteins as identified by Genome Ontology annotations. Consistent with the prostate fibroblast ageing data set, CXCL12 was one of the significantly upregulated genes with senescence. Co-cultures of prostate epithelium with senescent fibroblasts produced significantly higher epithelial cell proliferation rates when compared with pre-senescent fibroblasts. Conditioned medium from the senescent fibroblasts recapitulated these findings, indicating that a large component of the growth-promoting effect was due to paracrine-acting factors. Further work designed to identify individual genes contributing to the paracrine effects determined that fibroblast-derived amphiregulin directly contributed to the senescence-associated proliferative effects on prostate epithelium (Bavik *et al*, 2006).

Global studies of the prostate fibroblast senescence programme determined that specific categories of biological processes such as cell communication, extracellular matrix structural constituents, immune and inflammatory responses, and insulin-like growth factor binding were highly enriched. Many upregulated genes within these diverse functional categories could contribute to the effects of stromal senescence on epithelial cell behaviour. One set of such genes are secreted autocrine- or paracrine-acting growth factors including amphiregulin, hepatocyte growth factor, bone morphogenic protein 1, macrophage-inhibitory cytokine 1 (MIC-1/PLAB/GDF15), connective tissue growth factor, and vascular endothelial growth factor (VEGF). The insulin-like growth factor binding proteins were also significantly altered by senescence, including IGFBP2, IGFBP3, IGFBP5, and IGFBP6. These proteins may directly signal through prostate epithelial surface receptors, or may also signal through crosstalk to the androgen pathway, which is central to prostate carcinogenesis (Culig *et al*, 1994). It is well-established that VEGF expression is important in angiogenesis, an essential prerequisite for tumour growth beyond hypoxia-limiting sizes (Hrouda *et al*, 2003). Vascular endothelial growth factor also serves as a chemo attractant for tumour-associated macrophages, which further promote tumorigenesis and progression (Lewis and Pollard, 2006).

A second category of genes upregulated in senescent fibroblasts includes chemokines and cytokines; CXCL12, CXCL1, CCL11, CCL13, CCL20, C17, IL6, and IL8. As with the growth factors, these proteins, CXCL12, CXCL1, IL6, and IL8 in particular, have previously been shown to stimulate the growth, survival, or invasive behaviour of prostate epithelium (Engl *et al*, 2006). In addition, the cytokines IL8 and C17 are angiogenic (Hrouda *et al*, 2003). It has been increasingly recognised that inflammation in general, and macrophages in particular, may play significant roles in prostate carcinogenesis and cancer progression. Chemokines secreted by senescent stroma such as CXCL1 and IL8 may attract macrophages, and subsequently modulate their function; for example, converting anti-infectious M1 into the pro-carcinogenic M2 phenotype (Mantovani *et al*, 2004). M2 macrophages have been shown to secrete their own array of signalling molecules, which directly stimulate the epithelium, cause further inflammatory stimulation, and promote angiogenesis (Mantovani *et al*, 2006). M2 macrophages also have a diminished capacity for antigen presentation, which would promote the survival of neoplastic clones (Mantovani *et al*, 2004).

The third set of upregulated genes in senescent stroma with the potential to modulate prostate carcinogenesis include extracellular matrix proteins, proteases, and protease inhibitors, which can serve to modify paracrine-acting proteins and remodel structural components of the microenvironment. Upregulated extracellular matrix proteins include collagen I*α*2, collagen III*α*1, collagen IV*α*5, collagen VI*α*1, collagen VI*α*2, collagen VII*α*1, collagen XV*α*1, laminin *α*4, laminin *β*2, integrin *α*5, integrin *β*1, integrin *β*4, osteonectin (SPARC), osteocalcin, osteopontin, syndecan 2, and fibronectin 1. Collagen IV is known to be upregulated in the *neu*-transformed, tumorigenic PC3 prostate cancer cell line, and osteopontin and osteocalcin are upregulated as LNCaP cells progress to the more aggressive C4-2 cell line (Stewart *et al*, 2004). Extracellular matrix-modifying proteinases and proteinase inhibitors upregulated with senescence include ADAMTS1, MMP2, MMP3, MMP9, cathepsin D, cathepsin O, cystatin S, cystatin B, cystatin C, TIMP1, and TIMP2. Matrix metalloproteinases can function to degrade the stroma and basement membrane, and aspects of invasive, and metastatic features of prostate cancers appear to be dependent on the ratios of MMP2, MMP9, and TIMP1 (Stewart *et al*, 2004). As many signalling molecules directly associate with components of the extracellular matrix, MMP-mediated alterations in the structural constituents of the microenvironment might affect the binding and availability of these factors. Matrix metalloproteinases also act to directly process growth factors, growth factor receptors, cytokines, chemokines, and other precursor proteins. Together, the sheer number of changes in the expression of microenvironment constituents that associate with the senescence gene expression programme provides challenges in determining which alteration represent the dominant influences on adjacent cell types (Figure 2). However, establishing the key nodes that dictate stromal effects on cellular characteristics such as proliferation, resistance to cell death, and invasive capacity, would serve to prioritise methods for interfering with detrimental signals.

## CLINICAL IMPLICATIONS AND FUTURE DIRECTIONS FOR INVESTIGATION

Current research focuses on identifying factors that underlie the profound age-associated increases in prostate cancer incidence. Clinically, the diagnosis and treatment of prostate cancer could be impacted at several levels. First, the identification of individual or patterns of secreted factors might provide biomarkers for early detection of prostate cancers, or more importantly, those cancers with the propensity for lethal behaviour. While prostate-specific antigen has been widely used as a screening test, its use and application continues to be controversial in many circles due to its lack of specificity and sensitivity. Novel patterns of secreted proteins identified through studies of ageing may provide diagnostic tools with the potential enhanced sensitivity by focusing on high-risk populations. In this context, it is important to recognise that molecular ageing may be distinct from chronological ageing, and senescence changes in the tumour microenvironment may reflect a particular sensitivity to DNA damaging agents or exposure to chronic inflammatory insults.

Second, prostate cancer has a significant lag time between the age at which cancers can be histologically detected with high prevalence and the age at which most clinical diagnoses are made. For example, pre-neoplastic PIN lesions can be identified in a substantial number (9%) of men aged 20–29, and asymptomatic histologic cancers and PIN lesions are reported to be present in 27 and 20% of men aged 30–39, respectively (Sakr *et al*, 1993). By contrast, only 0.01% of men aged less than 40 will be clinically diagnosed with prostate cancer (Jemal *et al*, 2007). Chemopreventive agents, potentially targeting cancer-promoting stromal factors altered in the context of ageing, administered during the lag times between PIN, cancer development, and/or progression to clinically-relevant disease, could delay or reverse the progression of prostate cancer.

Third, prostate cancer therapy may be improved by broadening the diversity of targets. In particular, directing treatment towards mechanisms modulating resistance to cytotoxic chemotherapy, or influencing components of the tumour microenvironment could offer novel combinations that may serve to abort resistance mechanisms and mitigate toxicities. For example, it is now possible to directly inhibit angiogenesis in the tumour microenvironment through approaches that modulate the effects of VEGF, a growth factor shown to be upregulated by senescent fibroblasts. Other agents such as COX-2 inhibitors may serve to influence effectors of the inflammatory or wound responses that exert growth or survival effects towards tumour cells. In prostate cancer, these efforts are not mature to date. However, improvements in our understanding of the biology of ageing and senescence should provide mechanistic insights into the relationship between these processes and neoplastic growth, and allow focused efforts to develop tools for the optimised preventive, diagnostic, and treatment strategies.

## Figures and Tables

**Table 1:** A concise sampling of genes with altered expression in senescence

**Gene**	**Gene name**	**Fold increase in senescence**	**Reference for effects in prostate cancer**
CXCL12	Stromal cell-derived factor 1	3.39	Engl *et al*, 2006
VEGF	Vascular endothelial growth factor	1.44	Hrouda *et al*, 2003; Lewis and Pollard, 2006
CXCL1	Melanoma growth stimulating activity *α*	3.55	Engl *et al*, 2006; Mantovani *et al*, 2004
IL6	Interleukin 6	2.31	Engl *et al*, 2006
IL8	Interleukin 8	6.76	Hrouda *et al*, 2003; Mantovani *et al*, 2004; Engl *et al*, 2006
COL4A5	Collagen 4 *α* 5	2.34	Stewart *et al*, 2004
SPARC	Osteonectin	1.92	Stewart *et al*, 2004
SPP1	Osteopontin	1.20	Stewart *et al*, 2004
MMP2	Matrix metalloproteinase 2	4.33	Stewart *et al*, 2004
MMP9	Matrix metalloproteinase 9	1.12	Stewart *et al*, 2004
TIMP1	Tissue inhibitor of metallopeptidase 1	2.65	Stewart *et al*, 2004
AREG	Amphiregulin	4.4	Bavik *et al*, 2006

## References

[bib1] Bavik C, Coleman I, Dean JP, Knudsen B, Plymate S, Nelson PS (2006) The gene expression program of prostate fibroblast senescence modulates neoplastic epithelial cell proliferation through paracrine mechanisms. Cancer Res 66: 794–802 16424011 10.1158/0008-5472.CAN-05-1716

[bib2] Begley L, Monteleon C, Shah RB, Macdonald JW, Macoska JA (2005) CXCL12 overexpression and secretion by aging fibroblasts enhance human prostate epithelial proliferation *in vitro*. Aging Cell 4: 291–298 16300481 10.1111/j.1474-9726.2005.00173.x

[bib3] Bhowmick NA, Chytil A, Plieth D, Gorska AE, Dumont N, Shappell S, Washington MK, Neilson EG, Moses HL (2004) TGF-beta signaling in fibroblasts modulates the oncogenic potential of adjacent epithelia. Science 303: 848–851 14764882 10.1126/science.1090922

[bib4] Chen Z, Trotman LC, Shaffer D, Lin HK, Dotan ZA, Niki M, Koutcher JA, Scher HI, Ludwig T, Gerald W, Cordon-Cardo C, Pandolfi PP (2005) Crucial role of p53-dependent cellular senescence in suppression of Pten-deficient tumorigenesis. Nature 436: 725–730 16079851 10.1038/nature03918 PMC1939938

[bib5] Collado M, Gil J, Efeyan A, Guerra C, Schuhmacher AJ, Barradas M, Benguria A, Zaballos A, Flores JM, Barbacid M, Beach D, Serrano M (2005) Tumour biology: senescence in premalignant tumours. Nature 436: 642 16079833 10.1038/436642a

[bib6] Culig Z, Hobisch A, Cronauer MV, Radmayr C, Trapman J, Hittmair A, Bartsch G, Klocker H (1994) Androgen receptor activation in prostatic tumor cell lines by insulin-like growth factor-I, keratinocyte growth factor, and epidermal growth factor. Cancer Res 54: 5474–5478 7522959

[bib7] Cunha GR, Hayward SW, Wang YZ, Ricke WA (2003) Role of the stromal microenvironment in carcinogenesis of the prostate. Int J Cancer 107: 1–10 12925950 10.1002/ijc.11335

[bib8] Dimri GP, Lee X, Basile G, Acosta M, Scott G, Roskelley C, Medrano EE, Linskens M, Rubelj I, Pereira-Smith O, Peacocke M, Campisi J (1995) A biomarker that identifies senescent human cells in culture and in aging skin *in vivo*. Proc Natl Acad Sci USA 92: 9363–9367 7568133 10.1073/pnas.92.20.9363 PMC40985

[bib9] Engl T, Relja B, Blumenberg C, Muller I, Ringel EM, Beecken WD, Jonas D, Blaheta RA (2006) Prostate tumor CXC-chemokine profile correlates with cell adhesion to endothelium and extracellular matrix. Life Sci 78: 1784–1793 16263140 10.1016/j.lfs.2005.08.019

[bib10] Herbig U, Ferreira M, Condel L, Carey D, Sedivy JM (2006) Cellular senescence in aging primates. Science 311: 1257 16456035 10.1126/science.1122446

[bib11] Hrouda D, Nicol DL, Gardiner RA (2003) The role of angiogenesis in prostate development and the pathogenesis of prostate cancer. Urol Res 30: 347–355 12599013 10.1007/s00240-002-0287-9

[bib12] Jemal A, Siegel R, Ward E, Murray T, Xu J, Thun MJ (2007) Cancer statistics, 2007. CA Cancer J Clin 57: 43–66 17237035 10.3322/canjclin.57.1.43

[bib13] Krishnamurthy J, Torrice C, Ramsey MR, Kovalev GI, Al-Regaiey K, Su L, Sharpless NE (2004) Ink4a/Arf expression is a biomarker of aging. J Clin Invest 114: 1299–1307 15520862 10.1172/JCI22475 PMC524230

[bib14] Krtolica A, Parrinello S, Lockett S, Desprez PY, Campisi J (2001) Senescent fibroblasts promote epithelial cell growth and tumorigenesis: a link between cancer and aging. Proc Natl Acad Sci USA 98: 12072–12077 11593017 10.1073/pnas.211053698 PMC59769

[bib15] Lewis CE, Pollard JW (2006) Distinct role of macrophages in different tumor microenvironments. Cancer Res 66: 605–612 16423985 10.1158/0008-5472.CAN-05-4005

[bib16] Mantovani A, Allavena P, Sozzani S, Vecchi A, Locati M, Sica A (2004) Chemokines in the recruitment and shaping of the leukocyte infiltrate of tumors. Semin Cancer Biol 14: 155–160 15246050 10.1016/j.semcancer.2003.10.001

[bib17] Mantovani A, Schioppa T, Porta C, Allavena P, Sica A (2006) Role of tumor-associated macrophages in tumor progression and invasion. Cancer Metastasis Rev 25: 315–322 16967326 10.1007/s10555-006-9001-7

[bib18] Melk A, Schmidt BM, Takeuchi O, Sawitzki B, Rayner DC, Halloran PF (2004) Expression of p16INK4a and other cell cycle regulator and senescence associated genes in aging human kidney. Kidney Int 65: 510–520 14717921 10.1111/j.1523-1755.2004.00438.x

[bib19] Michaloglou C, Vredeveld LC, Soengas MS, Denoyelle C, Kuilman T, van der Horst CM, Majoor DM, Shay JW, Mooi WJ, Peeper DS (2005) BRAFE600-associated senescence-like cell cycle arrest of human naevi. Nature 436: 720–724 16079850 10.1038/nature03890

[bib20] Paradis V, Youssef N, Dargere D, Ba N, Bonvoust F, Deschatrette J, Bedossa P (2001) Replicative senescence in normal liver, chronic hepatitis C, and hepatocellular carcinomas. Hum Pathol 32: 327–332 11274643 10.1053/hupa.2001.22747

[bib21] Parrinello S, Coppe JP, Krtolica A, Campisi J (2005) Stromal-epithelial interactions in aging and cancer: senescent fibroblasts alter epithelial cell differentiation. J Cell Sci 118: 485–496 15657080 10.1242/jcs.01635 PMC4939801

[bib22] Roberson RS, Kussick SJ, Vallieres E, Chen SY, Wu DY (2005) Escape from therapy-induced accelerated cellular senescence in p53-null lung cancer cells and in human lung cancers. Cancer Res 65: 2795–2803 15805280 10.1158/0008-5472.CAN-04-1270

[bib23] Roninson IB (2003) Tumor cell senescence in cancer treatment. Cancer Res 63: 2705–2715 12782571

[bib24] Sakr WA, Haas GP, Cassin BF, Pontes JE, Crissman JD (1993) The frequency of carcinoma and intraepithelial neoplasia of the prostate in young male patients. J Urol 150: 379–385 8326560 10.1016/s0022-5347(17)35487-3

[bib25] Schmitt CA, Fridman JS, Yang M, Lee S, Baranov E, Hoffman RM, Lowe SW (2002) A senescence program controlled by p53 and p16INK4a contributes to the outcome of cancer therapy. Cell 109: 335–346 12015983 10.1016/s0092-8674(02)00734-1

[bib26] Statistics O.f.N. (2006) Cancer Statistics Registrations: Registrations of Cancer Diagnosed in 2004, England. Series MB1 no. 35 London: National Statistics

[bib27] Stewart DA, Cooper CR, Sikes RA (2004) Changes in extracellular matrix (ECM) and ECM-associated proteins in the metastatic progression of prostate cancer. Reprod Biol Endocrinol 2: 2 14711377 10.1186/1477-7827-2-2 PMC320496

[bib28] te Poele RH, Okorokov AL, Jardine L, Cummings J, Joel SP (2002) DNA damage is able to induce senescence in tumor cells *in vitro* and *in vivo*. Cancer Res 62: 1876–1883 11912168

[bib29] Tuxhorn JA, McAlhany SJ, Dang TD, Ayala GE, Rowley DR (2002) Stromal cells promote angiogenesis and growth of human prostate tumors in a differential reactive stroma (DRS) xenograft model. Cancer Res 62: 3298–3307 12036948

[bib30] Yang G, Rosen DG, Zhang Z, Bast Jr RC, Mills GB, Colacino JA, Mercado-Uribe I, Liu J (2006) The chemokine growth-regulated oncogene 1 (Gro-1) links RAS signaling to the senescence of stromal fibroblasts and ovarian tumorigenesis. Proc Natl Acad Sci USA 103: 16472–16477 17060621 10.1073/pnas.0605752103 PMC1637606

[bib31] Zhang H (2007) Molecular signaling and genetic pathways of senescence: Its role in tumorigenesis and aging. J Cell Physiol 210: 567–574 17133363 10.1002/jcp.20919

